# Off-target interactions in the CRISPR-Cas9 Machinery: mechanisms and outcomes

**DOI:** 10.1016/j.bbrep.2025.102134

**Published:** 2025-07-05

**Authors:** Lyubov Yu. Kanazhevskaya, Polina V. Zhdanova, Alexander A. Chernonosov, Vladimir V. Koval

**Affiliations:** a Institute of Chemical Biology and Fundamental Medicine, Siberian Branch of the Russian Academy of Sciences, 630090 Novosibirsk, Russia; b Department of Natural Sciences, Novosibirsk State University, 630090 Novosibirsk, Russia

**Keywords:** CRISPR, Cas9, Genome editing, sgRNA, Mismatch tolerance, Off-target activity, Enzyme specificity

## Abstract

The in vivo editing of genetic information necessitates tools of unprecedented accuracy. CRISPR-Cas-based systems have emerged as leading technologies for precisely targeting the genome. The Cas9 endonuclease derived from *Streptococcus pyogenes* is the most commonly used instrument for targeted DNA cleavage. The development of engineered and chimeric Cas9 variants with enhanced activity and specificity has enabled not only the simple knockout of target genes but also the sophisticated engineering of the epigenome. This advancement has broadened the potential applications of CRISPR-Cas9 technology for the treatment of various disorders characterized by a combination of mutations, deletions, and duplications in the coding and non-coding regions of the genome. The inherent simplicity and predictability of the CRISPR-Cas9 targeting mechanism have led to an explosive growth in the development of prototype gene-editing drugs. However, their therapeutic application is still challenged by potential off-target effects. The erroneous editing of tumor suppressors and oncogenes could lead to adverse outcomes that mitigate the benefits of CRISPR therapy. The evolution of DNA-targeting technologies requires a comprehensive understanding of the mechanisms underlying CRISPR-Cas9 off-target binding and cleavage. The use of massive libraries of DNA targets and guide RNAs, coupled with high-throughput sequencing, contributes significantly to the analysis of mismatch tolerance. Nevertheless, the detection of ultra-low levels of off-target activity is hindered by the sensitivity limitations of current technologies. This review focuses on the mechanisms responsible for off-target interactions during CRISPR-Cas9-mediated gene editing. We discuss the influence of various factors, including nucleotide context, enzyme concentration, guide RNA structure, and the energetics of the RNA–DNA hybrid on mismatch tolerance in vitro and in vivo. Recent advances in the development of technologies for predicting off-target effects are briefly summarized. Particular emphasis is placed on the role of the Cas9 protein structure in the allosteric regulation of the specific and non-specific activity of the Cas9–sgRNA complex.

## Highlights

• The off-target activity of CRISPR/Cas9 is influenced by mismatch position, DNA context and the guide RNA secondary structure.
• The evolution of DNA targeting technologies requires methods for detecting low-level non-specific activity of Cas9/sgRNA.
• Comprehensive understanding the mechanism of mismatch tolerance can be advantageous in genetic engineering applications.

## 1 Introduction

CRISPR-Cas9 (clustered regularly interspaced short palindromic repeats (CRISPR)-associated protein 9)-based DNA editing systems are innovative tools used to manipulate genetic information. Originally discovered as a defense mechanism against foreign DNA in bacteria and archaea [1,2], the CRISPR-Cas9 system has rapidly evolved into a technology for editing genes in almost any living organism [3,4]. The most widely used Type II CRISPR-Cas system in combination with Cas9 nuclease includes the minimal set of elements required for editing: Cas9 endonuclease, CRISPR-associated RNA (CRISPR RNA, crRNA), *trans*-activating crRNA (tracrRNA), and ribonuclease III (RNase III) [5,6]. The multidomain structure of Cas9 allows the enzyme to independently detect, bind, and cleave both strands of foreign DNA [4,7]. To target a specific DNA locus, Cas9 binds to a crRNA-tracrRNA duplex containing a guide sequence complementary to the target (protospacer) at its 5′-end. A double-strand break (DSB) can only be introduced into the protospacer sequence adjacent to the PAM motif (protospacer adjacent motif, 5'-NGG-3' for SpCas9 from *Streptococcus pyogenes*) [8]. The further repair of DSB is accomplished either by error-prone non-homologous end joining (NHEJ) or by high-fidelity homology-directed repair (HDR) in the presence of template DNA [9]. The NHEJ mechanism usually results in insertions, deletions, stop codons, frame shifts, and the subsequent attenuation of the unwanted gene. The HDR, in turn, is a simple method for introducing small and manageable changes to a gene. Cas9-based gene-editing tools commonly utilize an artificial chimeric single-stranded guide RNA (single guide, sgRNA) that retains a 20-nucleotide guide sequence of crRNA at the 5′-end and hairpin structures of the tracrRNA at the 3′-end [4]. Numerous studies have proven the efficacy of genomic DNA targeting using the Cas9-sgRNA complex, but the selectivity of this effect is highly variable, hampering the translation of CRISPR-Cas9 technology into a routine laboratory procedure [[10], [11], [12]].

**Fig. 1:**
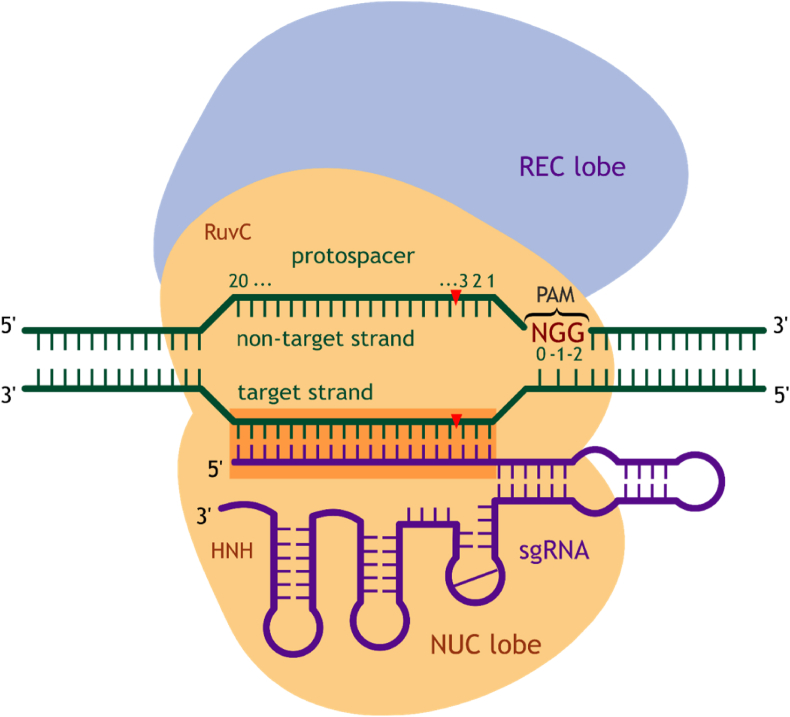
Schematic representation of the Cas9-sgRNA complex with the target DNA. The guide RNA (purple) is hybridized with its guide sequence (orange) with the complementary strand of the target DNA (green). The nucleotides of the target DNA sequence are numbered toward the 5'-end, starting from the PAM.

The topology of the sgRNA-DNA heteroduplex complexed to Cas9 nuclease involves the hybridization of the RNA guide sequence and the 20 nt protospacer at the 3'-end of PAM (Fig. 1). In principle, the specificity of Cas9 should be strongly limited by the 20 nt guide sequence and the presence of the PAM trinucleotide, but, in practice, off-target binding and cleavage is observed even for DNA sequences with a three–five base pair mismatch in the PAM-distal region of the protospacer [13,14], whereas off-target binding is possible in the presence of 12-15 mismatches [10,13]. The gain in editing efficiency often weakens the specificity and selectivity of the system, because an increase in the specific activity of the ribonucleoprotein (RNP) complex correlates with an increase in its non-specific activity. A well-designed sgRNA directed against a target locus has one perfect match in the genome and several sites with single or double mismatches genome-wide. However, if one considers the targets with more than three mismatches, the spectrum of targets would number in the thousands. At present, the off-target activity of the CRISPR-Cas9 system is one of the major challenges to the safe therapeutic application of this technology.

Over the past decade, the effect of mismatches, insertions, and deletions in each position of the protospacer sequence and the PAM motif on the efficiency of CRISPR-Cas-mediated hydrolysis has been extensively studied. Early studies found that the hydrolytic activity of the RNP complex is most sensitive to mismatches in the PAM-proximal end of the protospacer (seed region, positions 1–8), while the presence of mismatches in the PAM-distal region (positions 18–24) barely interferes with the accumulation of the hydrolyzed product [4,[14], [15], [16]]. It has been further shown that the entire twenty-base pair target sequence and two-base pair PAM can contribute to the Cas9 specificity [[17], [18], [19]]. Importantly, results obtained for different targets and different cell types, both in vitro and in vivo, are often not consistent with each other, indicating the complex and multi-parameter nature of the interactions between Cas9-sgRNA and the target DNA. It is widely accepted that non-specific cleavage by Cas9 depends on the mismatch position and nucleotide context, the guide RNA secondary structure and length, the local chromatin accessibility, and the concentration of nuclease [[20], [21], [22]]. Comprehensive studies have elucidated biophysical processes underlying the sensitivity and specificity of the CRISPR-Cas9 system [19,[23], [24], [25]]. It is assumed that the non-specific activity is caused by failures in particular checkpoints of the Cas9 multistep mechanism. Nevertheless, principles of sequence-dependent efficacy across guide sequences and sequence-dependent sensitivity to sgRNA-DNA target mismatches are less comprehensively addressed. In addition, the method by which Cas9 and its guide RNA are delivered to genomic DNA are of major importance for the efficiency and specificity (reviewed in Ref. [26]). Many tools have been developed to computational and experimental prediction of potential off-target sites (reviewed in Ref. [27]); however, building predictive biophysical models for off-target activity on a case-by-case basis remains a challenge.

Today, the development of base-editing technologies facilitate the simultaneous correction of many mutations or accurate nucleotide changes, leading to further advances in the development of multiplex editing tools and base-editing technology fiction [28]. The principal therapeutic applications of CRISPR-Cas systems involve precise DNA editing to correct pathogenic mutations in hereditary disorders [12,29]. CRISPR-Cas nucleases, such as Cas9, Cas12, and Cas13, are being actively investigated for a range of pathologies, including cystic fibrosis [30], hemophilia B [31,32], and sickle cell disease [33]. In cancer therapy, CRISPR/Cas technology is leveraged for functional genomics screening, diagnostics, and therapeutic interventions. Treatment approaches involve targeting cancer-associated genes, enhancing the efficacy and safety of chimeric antigen receptor T-cells through gene editing [34,35], and modulating gene expression using CRISPR interference (CRISPRi) and CRISPR activation (CRISPRa) techniques [34,36]. In addition to genome engineering, the CRISPR toolbox could potentially be used to prevent and treat infectious diseases by targeting viral genomes, such as those of HIV-1 and hepatitis B virus [29,37,38], as well as for combating bacterial antibiotic resistance [39,40]. Furthemore, CRISPR-Cas technologies are being adapted to address diseases with complex etiologies by targeting non-coding RNAs [41], modulating gene expression in long QT syndrome and facioscapulohumeral muscular dystrophy [[42], [43], [44]], reducing the expression of mutant Huntington alleles, and altering the expression of genes involved in autoimmune diseases [36,45]. These wide-ranging applications underscore the transformative potential of CRISPR-based technologies in precision medicine by enabling targeted and potentially curative therapies across a broad spectrum of human diseases [12,36]. CRISPR-Cas technology requires the careful control of both the accuracy and precision of genome editing in clinical settings and ultimately aims to reduce or eliminate undesired events by controlling target site recognition and DNA repair outcomes.

This review focuses on mechanisms underlying off-target binding and cleavage by Cas9 from *Streptococcus pyogenes* (except where otherwise specified). We aim to discuss the effect of mismatches throughout the sgRNA-DNA target interface, as well as the role of guide RNA secondary structure and sequence in the Cas9 specificity. We also explore the influence of structural features and allosteric regulation of the Cas9-sgRNA complex on the non-specific activity. Attention is paid to the basis of enhanced specificity of modern engineered Cas9 variants. Keeping in line with the selected direction of the discussion, we have omitted the effect of delivery strategies on nuclease specificity out of consideration.

## 2 Molecular mechanisms of the Cas9-sgRNA complex activity

Targeting of the Cas9-sgRNA complex is initiated by the recognition of a specific PAM sequence located in the non-target DNA strand (Fig. 1). The binding process is facilitated by interactions of PAM with a positively charged groove within the C-terminal domain between REC and NUC lobes of Cas9 [46,47]. This transient PAM surveillance step is independent of the downstream DNA sequence. The PAM–RNP interactions further destabilize the adjacent DNA duplex that is marked by a sharp kink turn in the target strand immediately upstream of the PAM [48]. In presence of a protospacer, the subsequent melting of the DNA duplex and formation of the RNA-DNA heteroduplex occur across the entire target through a stepwise unwinding mechanism, with the PAM motif serving as an allosteric regulator of Cas9 activation [49]. An elegant experiment with single-stranded DNA (ssDNA) targets corroborates this mechanism [50]. When the protospacer was adjacent to the PAM complement rather than true PAM sequence, the cleavage rate is reduced by two orders of magnitude compared to normal dsDNA targets. The hybridization of ssDNA to a complementary strand whose sequence overlapped the PAM motif increased the rate of hydrolysis to its original level. This result indicates that PAM recognition plays a central role in target recognition.

Cas9 has a highly dynamic structure and undergoes multiple conformational rearrangements (e.g., “open-to-close” conformational transition) in the course of the target sequence validation and incision. Once the Cas9-RNA-DNA ternary complex is formed, the interaction between the RNA-DNA hybrid and the REC lobe can trigger the activation of the distally located catalytic domains in an allosteric manner [[51], [52], [53]]. The importance of RNA–DNA complementarity throughout the target region, rather than only the seed sequence closest to the PAM, in controlling Cas9 cleavage specificity has been confirmed experimentally [52,54,55]. In the inactive conformation of Cas9 nuclease domains HNH and RuvC are located more than 30 Å away from the cleavage site of target DNA strand [47,56]. The collective conformational dynamics of REC lobe domains were found to be a key regulator of the HNH domain movement and activation [57]. REC3 acts as an allosteric effector that recognizes the RNA–DNA heteroduplex and re-orients REC2, which enables HNH docking at the active site [54,58]. Computational studies also revealed that REC2 highly couples with HNH, suggesting its possible role in the allosteric transmission between REC3 and HNH [53]. When Cas9 is bound to off-target substrates, REC2 can sterically occludes HNH in the inactive “conformational checkpoint”, thus preventing the cleavage of an incorrect DNA sequence [51].

The cleavage of the target and non-target strand 3 bp downstream from the PAM is performed by the HNH and RuvC nuclease domains, respectively [4]. There is a tight correlation between RuvC domain cleavage activity and the presence of an activated HNH conformational state [52]. It was shown that an allostery between the HNH and RuvC domains is ensured by two hinge regions: L1 (residues 765–780) and L2 (residues 906–918) [46]. These linkers undergo a striking rearrangement in response to the reorientation of the HNH domain, thus providing RuvC activity for accurate DNA cleavage. As long as DSB is formed, Cas9 remains tightly bound to both ends of the cleaved DNA for hours until repair/replication, as reported by in vitro and in vivo studies [[59], [60], [61]]. The long lifetime of the ternary complex makes Cas9 a single-turnover enzyme and reduces the rate of subsequent DSB processing. A single-molecule approach showed that the 3' flap generated by the cleaved non-target strand can be digested by exonucleases due to the solvent exposure [62]. It was suggested that, under cellular conditions, this exposed 3′ end can recruit an ssDNA-targeting motor protein, such as helicase, polymerase, or histone chaperone, to promote Cas9 dissociation and the repair of DSB [63,64]. Despite extensive control and proofreading checkpoints, the CRISPR–Cas9 machinery may be erroneous, resulting in off-target cleavage. The mechanisms of non-specific activity are sophisticated and not completely clear. Under various conditions, mismatched nucleotides can be tolerated by Cas9, can block the target cleavage, or can even enhance the RNP complex's activity. The following sections discuss multiple factors and mechanisms that influence the outcomes of Cas9-sgRNA targeting.

## 3 Tolerance to mismatches in key regions of the target DNA

Genomic DNA comprises sequences adjacent to the PAM motif that slightly deviate from the intended target and can be subjected to non-specific hydrolysis by the CRISPR-Cas9 system. The term “mismatch tolerance” characterizes the ability of Cas9 (or other genome editing nuclease) to cleave unintended sequences [4]. Mismatches located in different regions of the protospacer govern the outcome of Cas9-sgRNA activity. The detection of off-targets is of utmost importance, since even an ultra-low frequency of unintended mutations in the genome can be critical for in vivo editing. Early studies on the off-target activity of Cas9, both in vitro and in vivo, employed model DNA sequences such as short oligonucleotides or plasmids that mimic specific genomic loci. Editing efficiency was conventionally evaluated using the T7 endonuclease I (T7E1) assay, which detects mutations or insertions/deletions (indels) in DNA targets using PCR amplification with specific primers to on- and off-target sequences, followed by the endonucleolytic digestion of mutant heteroduplexes [65,66]. However, the findings from these studies have not always been consistent, potentially due to the limited sensitivity of the T7E1 assay, which only detects mutations occurring at frequencies greater than 1 %. For example, in the context of editing the *C4BPB* and *CCR5* genes in human cells, the T7E1 assay failed to identify any off-target activity of the Cas9-sgRNA complex [67]. The later study showed the presence of non-target mutations in this system at frequencies as low as 0.01 %–0.1 % using a deep-sequencing technique [15]. Such data stimulated the emergence of powerful methods based on deep-sequencing technology for the assessment of off-target cleavage by the RNP complex. To date, many methods employing next-generation sequencing (ChIP-seq, GUIDE-seq [68], SITE-seq [69], Digenome-seq [70], and others) for the comprehensive evaluation of editing efficiency and specificity become widespread in CRISPR-Cas9 research (reviewed in Ref. [71]).

### 3.1 PAM motif

**Fig. 2:**
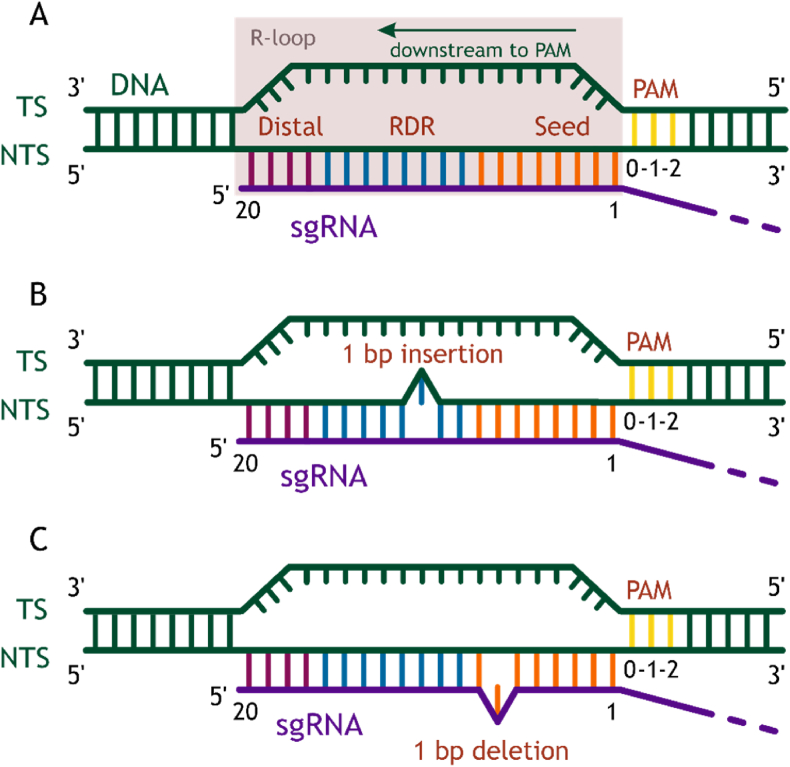
A detailed schematic representation of an RNA-DNA heteroduplex. (A) A completely unwound DNA duplex (green) in the form of R-loop is stabilized by sgRNA (purple) hybridization. Possible mismatch positions between sgRNA and target DNA are shown in orange (seed region), blue (RDR region), and plum (PAM-distal region) downstream to the PAM sequence (yellow). The R-loop is highlighted by a dusty rose-colored rectangle. (B) The presence of one non-complementary base (1 bp insertion) among matched bases of RNA-DNA heteroduplex results in the formation of a single-base DNA bulge. (C) The absence of one base relative to the protospacer sequence (1 bp deletion) results in the formation of an RNA bulge.

The specific recognition of the PAM sequence is thought to be essential for target-binding and the initiation of DNA unwinding in the course of R-loop formation (Fig. 2A) [4,7,15,16,72]. Identification of the potential target begins with the recognition of the PAM motif and the rapid dissociation of the RNP complex from non-PAM sites [50]. The nucleotide content and length of the PAM motif vary depending on the origin of the Cas9 nuclease [73]. The canonical PAM motif of a widely utilized Cas9 nuclease from *Streptococcus pyogenes* (SpCas9) is a trinucleotide sequence 5'-NGG-3' (where N refers to A, T, G, or C). However, it was shown that Cas9 displays a degree of tolerance to substitutions of certain bases within the PAM and can cleave sequences with 5'-NAG-3', 5'-NGA-3', and 5'-NCG-3' PAMs in vitro and in vivo, albeit less efficiently than 5'-NGG-3' [17,20,21,74]. The analysis of the binding efficiency of the catalytically inactive dCas9 variant (with D10A and H840A mutations) has demonstrated a slow association rate for mutant PAMs [18,75]. A longer equilibration time (12 h) resulted in the stabilization of the dCas9-sgRNA-DNA complex with NGA and NAG PAMs, suggesting that these sequences compete with the classical NGG motif. A comprehensive test of SpCas9 efficiency with non-canonical PAMs using a GFP-reporter system indicated that significant off-target mutagenesis can be induced by CRISPR-Cas9 with non-NGG PAMs in human cells [74]. Thus, a cleavage possibility for sequences attached to meaningful non-canonical PAMs should be taken into account in developing CRISPR-Cas9 editing tools. In SpCas9, the canonical 5′-NGG-3′ PAM is read out by a pair of arginine residues, Arg1333 and Arg1335, inserted into the major groove of the DNA duplex [47]. Depending on the specific application of CRISPR-Cas technology, reducing or increasing the genomic diversity of the target loci may be required. The engineered SpCas9 variants contain substitutions around positions 1333–1335 possess the ability to directly recognize the altered PAM nucleotides [76,77]. Exploring other Streptococci Cas9 orthologs that show high sequence identity with SpCas9 but harbor a different RxQ PAM-binding motif has promoted the further expansion of a library of available PAM sequences [[78], [79], [80]]. Moreover, the rational design of closely related Cas9 orthologs resulted in the creation of chimeric Cas9 nucleases with increased efficiency and robust activity [81,82].

### 3.2 Seed region

The binding of the PAM motif promotes destabilization and the subsequent melting of the adjacent seed region of the target (Fig. 2A). The complementarity between bases in the seed sequence of sgRNA and the protospacer (positions 1–8) is critical for the Cas9-mediated cleavage of the target in many cases [4,16,17,19,83]. Single RNA-DNA hybrid mismatches in the PAM-proximal region can completely block editing, but this is not true for all targets. For example, in the study by Y. Fu and colleagues, which selected three different regions of the EGFP gene as targets, only one target showed critical dependence on mismatches at positions 1–8, while the other two targets were efficiently cleaved despite mismatches at any position except 1, 2, and 3 [14]. A similar effect was observed for two-nucleotide mismatches located adjacent to or separated by normal base pairs. An analysis of a target library in the human clathrin light chain A gene (CLTA), containing all possible substitutions relative to the target sequence nucleotides, showed that, when single-nucleotide mismatches were assessed, bases 6–8 closest to PAM were generally highly specific, whereas the PAM-distal region displayed weak specificity under enzyme-limited conditions (100 nM). However, under enzyme-excess conditions (1000 nM), single mutations closest to PAM no longer inhibited cleavage, suggesting that the specificity of the seed region is reduced at high Cas9 concentrations [19]. The presence of two or more mismatches, if at least one is located in the seed region, is effectively discriminated by the RNP complex, thus preventing off-target activity [15,21]. The disruption of complementarity in the seed region likely causes mismatches in the early steps of sgRNA insertion, hindering efficient binding, as evidenced by data on the association kinetics of complexes with the catalytically inactive dCas9 variant [18,50,75]. The low binding rate of dCas9 is attributed to the rapid rejection of such targets after PAM-dependent initial association. The requirement for matching PAM-proximal versus -distal nucleotides is consistent with a model of unidirectional elongation of the RNA-DNA heteroduplex, starting at the PAM-proximal end. Some data suggest that sequence homology near the PAM is critical for initiating the unwinding of the target DNA duplex until the R-loop reaches a length of ∼12 nt [50]. Beyond this point, the energy required to extend the RNA-DNA heteroduplex is less than that needed for the reverse reaction. This concept was confirmed by studying the interaction of Cas9 with an unusual target that not only contained mismatches with guide RNA at positions 1–2, but also lacked complementarity at those positions, resulting in a small bubble in the DNA duplex. Such a substrate proved to be a strong competitor for binding to the RNP complex and was cleaved with efficiency close to the target sequence. Most likely, the presence of the bubble compensated for the energetic losses that occur when attempting to anneal the mismatched duplex next to the PAM motif. This allows Cas9-sgRNA to bypass mismatches and re-initiate the nucleation of the RNA-DNA hybrid downstream of the bubble, ensuring DNA–substrate strand separation [50]. If the mismatch with the guide RNA is within the first two nucleotides adjacent to the PAM, the RNP complex loses its ability to probe and recognize the remainder of the DNA, meaning that mismatches occurring early in the directed fusion process prematurely interrupt target interrogation.

In a study investigating the impact of mismatches on the efficiency of Cas9-mediated DNA hydrolysis within the pneumococcal bacteriophage system, it was determined that the extent of RNP complex activity inhibition in the presence of single-nucleotide seed mismatches is contingent upon the nature of the unpaired nucleotide [8,84]. Specifically, the introduction of a G→T substitution impeded target processing, whereas G→A and G→C substitutions enhanced Cas9 efficiency. This trend was consistent across various targets and systems, with transversion substitutions (Pu↔Py) causing a significantly greater reduction in editing efficiency compared to transition substitutions (Pu↔Pu, Py↔ Py) [17,24]. The presence of an rC:dC pair in the RNA-DNA hybrid is especially associated with the highest level of inhibition of SpCas9 activity [17,21,24]. Thus, the off-target activity of the CRISPR-Cas9 system is influenced not only by the presence of mismatches in the seed region but also by their nucleotide context. The influence of nucleotide nature on tolerance to mismatches may account for the observed variability in cleavage efficiency across different DNA targets [68]. Cas9-sgRNA targeting of various DNA loci, even under identical conditions, often exhibits divergent, and at times contradictory, patterns of mismatch tolerance [14,17,69,85]. Particularly, SITE-Seq sequencing of Cas9-sgRNA amplicons targeting regions of the *VEGFA* and *FANCF* genes revealed mutations at twenty-three and seven off-target sites, respectively, whereas only one off-target site was identified for the *PTPRC* gene [85]. Meanwhile, no off-target effects were observed for several highly specific targets when edited in cell culture [15]. When Cas9 aims at different regions of the same gene, the sensitivity of the RNP complex to protospacer mismatches may also fluctuate significantly [14,21]. Furthermore, specific mismatch-sensitive positions frequently differ between these targets. Therefore, massive profiling of targets through high-throughput sequencing is essential for quantifying the effects of nucleotide context and mismatch presence on the kinetics of sgRNA invasion into diverse DNA sequences.

### 3.3 PAM-distal region

PAM distal bases (positions 18–20) are generally considered to have less stringent requirements for complementarity with guide RNA, and therefore they pose a risk of off-target cleavage (Fig. 2A). Indeed, single- and double-nucleotide mismatches at positions 18–20 from the PAM typically do not interfere with Cas9-mediated hydrolysis [3,4,14,16]. However, when the number of mismatches increases to three or more, either consecutively or interspersed with matched pairs, the activity of the RNP complex is substantially diminished [14,19,86]. Notably, while a single-nucleotide mismatch in the PAM-distal region alone does not significantly impact cleavage efficiency, its presence alongside mismatches in the central and seed regions can inhibit Cas9-sgRNA activity [87]. Detailed mechanistic studies of the cleavage process have shown that mismatches in the PAM-distal region do not disrupt target binding, but can lead to the formation of a catalytically inactive complex [18,24]. These findings align with observations that not all non-target sequences bound to the RNP complex undergo cleavage [85,88]. The RNP complex maintains the ability to bind off-targets with 12-15 consecutive PAM-distal mismatches at the level of on-target DNA in vitro and in cell culture [13,88]. Kinetic studies on the binding of Cas9-sgRNA to a set of target and non-target DNA sequences reveal that, in case of seed region complementarity, mismatches in the PAM-distal region have minimal impact on the observed association constant, but significantly increase the rate of the complex dissociation [18]. The dissociation rate of a fully complementary complex is exceptionally low; however, a mismatch at position 16 leads to complete dissociation within 1 h. In other words, the dissociation rate of the Cas9-sgRNA-DNA complex is not dependent on seed region complementarity, but, rather, is influenced by the bases at the 5'-end of the target. Moreover, while PAM-distal mismatches alone do not affect dCas9 binding, they can significantly alter the association constant of dCas9 in the presence of additional mismatches on large time-scales [18,75]. Further study suggested that PAM-distal mismatches do not affect binding, but rather impair cleavage [89]. These data suggest a complex interplay of combinatorial perturbations in the target DNA sequence, affecting the process of RNA strand invasion in the course of specific complex formation.

From a structural perspective, the transition of Cas9 into a catalytically active conformation is accomplished by shifting the nuclease HNH domain toward the DNA, facilitating subsequent cleavage. The HNH domain itself lacks the ability to verify the target sequence, necessitating a specialized mechanism for this purpose. The substrate recognition domain REC3 interacts with the RNA-DNA heteroduplex and causes a change in the conformation of the domain REC2, which in turn mobilizes the HNH domain into its active conformation [58,90]. Since DNA cleavage demands significantly greater complementarity with the guide RNA than tight binding does [87,91], and the rate of strand cleavage correlates more with the melting of the DNA duplex than with binding affinity, it has been proposed that R-loop formation is crucial for transition of the RNP complex into its catalytically active state [54,92]. Single-molecule FRET analysis has demonstrated that the configuration of the melted R-loop is assessed by the REC3 domain and the RuvC-HNH interdomain linker, steering the process towards target hydrolysis [55]. Structural mechanisms that govern off-target effects in the presence of PAM-distal mismatches apparently involve unusual interactions between certain amino acids of the RuvC domain and the highly distorted duplex [93]. The unusual nucleic acid conformation is stabilized by RuvC and appears to facilitate the binding of the mismatched target. Multiple mismatches in the PAM-distal region disrupt DNA strand unwinding, inhibiting the conformational transition of the HNH domain and thus preventing the cleavage of non-specific targets. The changing balance between the DNA duplex unwinding and rewinding induced by mismatches creates an energetic competition between the annealing of the target strand with sgRNA or with the non-target strand (rewiding). A similar mechanism appears to underlie the enhanced specificity of modified high-fidelity Cas9 variants such as EngCas9, HypaCas9, and Cas9-HF1, where mutations promote the rewinding of the DNA duplex and dissociation of RNP [55,89,94]. A successful hybridization of 8 bp RNA-DNA heteroduplex results in formation a stable ternary complex, regardless of mismatches in the PAM-distal region [13]. The wild-type Cas9 protein is unable to rapidly reject or cleave sequences with disrupted complementarity in the PAM-distal region, thereby limiting the rate of genome editing. This effect increases the minimal amount of Cas9-sgRNA required for editing, which, in turn, may lead to an increase in off-target cleavage events. Thus, while mismatches in the PAM motif and the seed region regulate the initial kinetics of dCas9 binding, the role of PAM-distal region complementarity cannot be overlooked when thoroughly considering the process of RNP complex integration into the DNA strand.

### 3.4 The central part of the protospacer (RDR area)

Another crucial protospacer region, known as the "reversibility-determining region" (RDR), spanning nucleotides 8 to 17 downstream of the PAM (Fig. 2A), has been identified by some researchers [18]. Investigations into the binding and dissociation kinetics of dCas9 with a library of specific and non-specific DNA sequences have shown that the dCas9-sgRNA-DNA complex dissociates rapidly in the presence of mismatches beyond the seed region, particularly at nucleotides 12–17. The dissociation rate of the complex increases proportionally with the number of mismatches. The study suggests that, if sgRNA manages to bypass mismatches in the PAM-proximal region, the complex may stabilize with non-target DNA due to complementarity in the PAM-distal region. Conversely, R-loop formation in the presence of RDR mismatches can undermine the long-term stability of the dCas9-sgRNA-DNA complex, even with perfect complementarity in the seed. Mismatches near the RDR affect the energy barrier to dissociation, allowing the RNA-DNA hybrid to dissociate more quickly. Homology of protospacer nucleotides 13 to 18 is critical not only for specific DNA recognition but also for cleavage [17,75]. Cryo-electron microscopy analyses of Cas9-sgRNA complexes with DNA targets containing three consecutive mismatches corroborate these findings [95,96]. For targets with mismatches at positions 12–14, short-term incubation with the ribonucleoprotein (RNP) complex for 1 min results in a linear conformation of the DNA duplex bound to the protein. Such a conformation refers to an intermediate state at the early stage of R-loop nucleation. With longer incubation times, such as 1 h, the DNA adopts a bent conformation, corresponding to the catalytically active state, suggesting that hydrolysis can occur but requires prolonged incubation with Cas9. The structure of the Cas9-sgRNA-DNA complex with mismatches at positions 15–17, regardless of the incubation time, demonstrated only the conformation with a linear form of the target DNA, indicating a complete lack of Cas9 activity on such DNA. As follows from structural analysis, there are no direct contacts between the protein domains and nucleotides 12–14, whereas positions 15–17 and 18–20 form multiple contacts with REC3. Thus, mismatches at positions 12–14 may evade discrimination by the REC3 domain, whereas mismatches further from the PAM can block nuclease activity.

Overall, the process of RNA-guided Cas9 interactions with a long DNA molecule in most cases represent a mixture of on- and off-target binding and incision events. It is known that the DNA-binding capacity identified by ChIP-seq and cleavage activity of the Cas9-sgRNA complex are not necessarily correlated [68,87,88,97]. The cleavage of many bound DNA targets is incomplete, so about 15 % of ternary complexes remain in a non-productive state for a long time [75,91]. Some targets undergo nicking on either target or non-target strand instead of dsDNA cleavage [98,99]. Some off-target sites can, in turn, be cleaved with high efficiency [17]. As has been shown for sgRNA targeting the Nanog gene, single seed mismatches at positions 5–10 stimulate effective DNA incision that surpasses incision at on-target sites [84,89]. This phenomenon is called “mismatch-activation”. Mismatch-activation results from the incomplete cleavage of the on-target sequence and the more complete cleavage of an off-target sequence. It was suggested that some mismatches allow DNA-bound complexes to escape from the inactive state, resulting in more complete cleavage. Since mismatch activation could favor off-target cleavage, it is important to consider this phenomenon when assessing the specificity of RNA.

## 4 Insertions/deletions in the RNA-DNA heteroduplex

CRISPR-Cas9 system off-targets are not exclusively limited to sequences with mismatches; they can also involve sequences characterized by the absence of one or more bases (deletions) (Fig. 2B) or the presence of one or more additional bases (insertions) (Fig. 2C). When sgRNA hybridizes with such targets, loops can form on either the target DNA strand or the guide RNA strand. Despite the significant deviations from the intended sequence, it has been shown that the RNP complex is capable of not only binding but also cleaving DNA with insertions and deletions [20,100]. Data on editing *HBB* and *CCR5* genes in HEK293T cells indicate a high level of tolerance to single-nucleotide loops [100]. In accordance with the general principles governing the impact of mismatches on Cas9 specificity, the efficiency of DNA hydrolysis in the presence of loops is influenced by the target's nature. This is partially attributable to the varying G/C content of the corresponding sgRNA sequences. High nuclease activity was observed in DNA with insertions at positions 1, 2, 7, 18, and 19, spanning all three key regions of the target. Models simulating a single-nucleotide deletion were largely resistant to Cas9-mediated hydrolysis if the deletion was located in the PAM-proximal region (positions 1–11). In other positions, on the contrary, the deletion enhanced the activity of the RNP complex, surpassing the hydrolysis levels of fully complementary sequences. This may be due to the enhanced flexibility of RNA molecules compared to DNA, which reduces the energy required for RNA-DNA hybrid formation in the presence of a single-nucleotide deletion. Model targets with deletions of 2-4 nt lengths were efficiently processed by the RNP complex, whereas similar insertions in the target DNA completely inhibited the activity of nuclease. The structures of off-target complexes reveal that the single-nucleotide deletions in these off-target substrates are not accommodated by bulging out the unpaired sgRNA nucleotide. Instead, the conformations of the sgRNAs remain largely unperturbed and the strand invasion proceeds over the unpaired bases to resume productive base-pairing downstream [93]. Notably, cleavage involving guide RNA with a bulge more frequently resulted in extended nucleotide deletions, while normal sgRNA typically led to 1-nucleotide insertions [100]. A recent kinetic study of various Cas9 variants interacting with a target library demonstrated that sequences containing deletions and insertions are generally processed more slowly than those containing mismatches. [85]. Deletions in the seed region of the target DNA prevent Cas9-sgRNA binding, but deletions located beyond the 9th nucleotide from the PAM still permit complex formation with moderate efficiency. It is likely that these deletions introduce additional steric constraints during R-loop formation. By considering CRISPR-Cas9 cleavage as an energy-driven process in which the efficiency significantly depends on the folding free energy changes, efficient and inefficient sgRNAs can be characterized by optimal and non-optimal values of the hybridization free energy change (ΔG_H_), respectively [101]. The preferential ΔG_H_ range may provide an explanation for the higher cleavage activity measured at bulged DNA targets.

## 5 Combinatorial effect of mismatches

The non-specific activity of the RNP complex is influenced by the position and nucleotide context of mismatches between the sgRNA and protospacer. However, these factors only partially account for the effects observed on actual genomic targets. Recent studies indicate that, in addition to the effects of specific mismatches, there is an intrinsic mismatch tolerance specific to each guide RNA [24,75]. More than 95 % of off-target sites identified in genomic experiments have two or more mismatches relative to the sgRNA sequence. Nevertheless, the factors driving the combinatorial effects of mismatches remain poorly understood due to insufficient experimental data. It was shown in the early study on the mechanisms ensuring the specificity of the CRISPR-Cas9 system that mismatches individually tolerated by Cas9 can preclude cleavage, if presented simultaneously in the amount of four [16]. Other studies have shown that off-target variants with G→A substitutions in the PAM motif and seed region tolerate single mismatches well, but result in a surprisingly severe reducing the cleavage efficiency when combined as double mismatches [17]. E. Boyle and colleagues examined how the productive binding energy of the RNP complex is altered in targets with double mismatches compared to their constitutive single mismatches [75]. Their data revealed that the effect of consecutive double mismatches was not merely additive. This effect was most pronounced at PAM-distal positions, where the energetic penalty for single-nucleotide mismatches was negligible (0 kT), whereas double mismatches incurred a penalty exceeding 1 kT. In contrast, nonconsecutive double mismatches that were at least 4 nt apart appeared additive. Thus, sufficiently distant mismatches have independent effects on productive binding. Interestingly, double mismatches separated by at least 4 nt were found to be additive, suggesting that sufficiently distant mismatches exert independent effects on binding. Mismatch combinations frequently exhibit complex epistatic-like behavior, making it difficult to apply simple additive models to analyze cleavage in the presence of higher-order mismatches [102]. For example, two mutations within the first or second 10 bases of a target reduce activity more significantly than expected from single mutations, whereas one mutation each in the two halves of the protospacer generally does not interact epistatically [24]. Consequently, the development of generalized rules for all guide RNAs without sacrificing accuracy remains challenging.

In the early theoretical model, the combinatorial effect of multiple mismatches was treated as "marginally independent" [20]. This model assumes that the tolerance for combined mismatches, in terms of off-/on-target cleavage ratio taken to equal the multiplied tolerances for individual mismatches. However, validation of the "marginal independent" model (for example, CFD score) using extensive sequence libraries revealed that it tends to overestimates off-target effects and provides an upper bound of the combinatorial effects [25]. In practice, many mismatch combinations are associated with much lower ratios of off-target to on-target activity (off-on ratio), indicating an “epistatic-like” effect. The degree of epistasis is estimated from the off-on ratios for a double-mismatched target in comparison to corresponding single-mismatched targets. The epistatic combinatorial effect can be explained by a kinetic model of Cas9-mediated DNA hydrolysis, in which the choice between R-loop formation and RNP complex dissociation prior to catalysis is determined by the relative energy barriers. When specific mismatch combinations result in elevated energy barriers, the catalytic cycle is blocked. R. Fu et al. proposed three quantitative rules to distinguish the combinatorial effects from those of individual mismatches [25]. Firstly, epistatic effects are more pronounced when mismatches cluster within the 10 nucleotides nearest to the PAM site or at the 19–20 positions in the PAM-distal region. Secondly, the proximity of mismatches to one another intensifies their mutual effect, with the strongest epistatic interactions occurring when mismatches are 1-6 nucleotides apart. Thirdly, adjacent mismatches exhibit minimal synergistic effects, forming small "bubbles" in the RNA-DNA hybrid that result in negligible changes to the overall energy barrier of base stacking. There is evidence suggesting that the synergistic effects of mismatches in the seed region of the target are due to the compensation of energy provided by the RNP complex during the sgRNA invasion [102]. This compensation enables the complex to bypass single mismatches in the PAM-proximal region. However, the energy barrier posed by two or more mismatches in the seed region cannot be sufficiently compensated, preventing the melting of the target DNA and allowing Cas9 to disengage. Consequently, the effects of multiple mismatches may be amplified, leading to a synergistic outcome. Current models for analyzing and predicting the nature of the mutual influence of different mutations and mismatches between sgRNA and the target hold significant promise for designing sgRNAs with customized activity, thereby enhancing allele-specific genome-editing capabilities [25,102].

## 6 The influence of sgRNA structure and sequence on Cas9 activity and specificity

The diversity in the biochemical behavior of Cas9 toward non-specific DNA is related not only to the specificity of the target but also to the structural configuration of sgRNA. Some sgRNAs exhibit high specificity and substantial alterations in DNA-binding affinity when exposed to non-specific sequences, while others demonstrate reduced sensitivity to mismatches, thereby decreasing the specificity of Cas9-mediated DNA cleavage [15,75]. In some cases, guide RNAs with overlapping sequences targeting the same genomic locus can vary in their efficacy [103]. Similarly to mismatches in DNA, sgRNA sequences may also contain diverse mismatches, and the effects of single-nucleotide mismatches in sgRNA sequences generally align with those observed for DNA mismatches [21,104]. By considering the complementarity between twenty nucleotides of the guide sequence and the non-target DNA strand, seed mismatches have the greatest detrimental impact on Cas9 cleavage efficiency, whereas unpaired nucleotides in the PAM-distal region pose less interference with the cleavage [21]. In a study focusing on CRISPR-mediated mutagenesis in Drosophila, intact sgRNAs were found to efficiently introduce DSBs in target DNA, whereas sgRNAs with one or two mismatches distal to the PAM motif exhibited significantly reduced hydrolysis efficiency [105]. The introduction of a third mismatch completely inhibited the activity of the RNP complex. Thus, target-mismatched sgRNAs with 1-2 PAM-distal mismatches can induce the potential off-target cleavage. The incorporation of nucleotides that mimic loops in the guide RNA diminished the hydrolytic capability of the RNP complex and could not result in off-target effects [20,100]. Interestingly, the occurrence of off-target activity is independent of whether a complex of CRISPR-associated (crRNA) and *trans*-activating RNA (tracrRNA) or a single chimeric sgRNA molecule is employed as a guide sequence [106].

The nucleotide composition of target-mismatched sgRNAs significantly affects its binding and cleavage efficiency. Large-scale assessments revealed that sgRNAs with a guanine base adjacent to the PAM (position 1, Fig. 2A) exhibited significantly higher mutagenesis frequency than other bases [104,107]. However, it is considered that the high efficiency of the Cas9-sgRNA complex can correlate with its lower specificity (ratio of the on-target vs. off-target indel frequency) [83,88]. For example, X. Wu et al. observed numerous off-target bindings of dCas9-sgRNA in the course of targeting promoters for all guide RNAs, excepting a few uracil-rich sgRNAs [88]. Authors have speculated that multiple uracils in the seed might induce the termination of sgRNA transcription by RNA polymerase, resulting in a reduced effective concentration of the Cas9-sgRNA complex within the cell. A lower abundance of sgRNA leads to lower editing efficacy at both on- and off-target sites. P. D. Hsu and colleagues found that mismatches involving rG:dT, rA:dC, and rC:dT pairs at positions 1–8 of the RNA-DNA hybrid were tolerated, whereas rC:dC and rU:dC pairs notably diminished the activity of the Cas9-sgRNA complex [21]. In addition, the G:U mispair in the PAM-proximal region should be avoided to ensure efficient genome editing [108]. A possible interpretation for the experimental observations has been provided by X-ray crystal structures of Cas9-sgRNA-DNA complexes with various mismatches in the guide RNA [56,93]. The seed region of RNA-DNA hybrid (positions 1–8) bound by the Cas9 protein usually adopts a distorted A-form stabilized by Watson–Crick base pairing. The structure of mismatched complexes indicated that the majority of nucleotide mismatches retain an intrahelical position within the RNA-DNA heteroduplex and are accommodated by non-canonical base-pairing interactions (wobble, Hoogsteen, and others). Cas9-sgRNA binding to the PAM-proximal mismatched DNA is accompanied by structural distortions of the target strand backbone, while the conformation of the gRNA backbone and base stacking within the seed region remain essentially unperturbed. This conclusion is consistent with the differential activity of Cas9 against off-targets containing the inverse mismatches rU:dG or rG:dT within the seed. While the rG:dT mismatch can overcome the energy barrier by positioning the bases in a "wobble"-geometry, adaptation of the rU:dG pair will be hindered due to increased rigidity. The formation of the rG:dT pair in the RNA-DNA hybrid is associated with the energetically favorable melting of the dT:dA pair in the DNA duplex, whereas the formation of the rU:dG pair requires the melting of the more stable dC:dG pair. The combination of these factors likely helps Cas9 to better discriminate rU:dG mismatches in the PAM-proximal region. It was suggested that off-target activity is largely determined by the kinetics and energetics of R-loop formation. Accordingly, an increased energetic penalty of base mispairing in the seed region prevents the efficient cleavage of such targets [93]. The tolerance to some seed mismatches can be related to the considerable conformational plasticity of Cas9, which enables it to compensate for local distortions via rearrangements in the REC2, REC3, and HNH domains [58]. In addition, Cas9 does not directly contact the RNA-DNA hybrid base pairs by means of the major- or minor-groove edges, lacking a steric mechanism to enforce Watson–Crick base pairing. Thus, for certain seed mismatches, moderate changes in the duplex width may have only minor effects on Cas9 interactions and activity.

Several studies have elucidated the direct correlation between the G/C content of the seed region of sgRNA and the efficiency of target editing [100,104,105,107,109]. It has been shown that a low G/C content, specifically when the occurrence is less than or equal to three, is associated with a reduced rate of mutations at edited loci [104,105]. Conversely, when guanine residues comprise more than 50 % of the sgRNA sequence and are concentrated in the seed region, the frequency of mutations increases [107]. The data obtained with the help of a free energy-based model for Cas9-gRNA target binding demonstrate that the gRNA with the GC-content of 70 % has an indel frequency of 30 % and a free energy change of sgRNA-DNA hybridization outside of the desired range [101,110]. Further analysis has suggested a nonlinear relationship between the percentage of G/C pairs in the guide sequence and cleavage efficiency, indicating that sgRNAs with excessively high or low G/C content are suboptimal. Thus, guide sequences in which the G/C percentage is in the range of 40–60 % can be considered favorable for editing efficiency [109]. The increased presence of guanine residues in the sgRNA sequence enhances both the on-target and off-target activity of Cas9. This is explained by the fact that G/C-rich RNA-DNA hybrids are more stable, also preventing dissociation of the RNP complex from off-target DNA [111].

The length and secondary structure of the guide RNA also significantly affect the efficiency and specificity of the CRISPR-Cas9 system [109,112,113]. The effect of sgRNA guide sequence secondary structure on Cas9 efficiency has been previously reported on in a contradictory manner. As suggested by X. Liu et al. [109], the ability of the sgRNA seed region to form secondary structures may raises the probability of target cleavage by stabilizing the Cas9-sgRNA complex. In contrast, another study indicated that a stable guide sequence secondary structure alone, but not the complete gRNA including the scaffold, is detrimental to Cas9 activity [113]. The sgRNAs with low self-folding stability exhibited much higher efficiencies, likely due its conformational plasticity and ability to invade the target DNA strand readily designing gRNAs [101]. X. Xu and colleagues have evaluated the correlation between the length of the RNA-DNA heteroduplex and the efficiency of target cleavage [114]. They found that RNA-DNA hybrids that are either too short (less than seven base pairs) or too long (greater than seventeen base pairs) correlate poorly with hydrolysis efficiency. In contrast, hybrids with lengths between 8 and 17 bp correlate with the highest editing efficiency. Therefore, for certain guide RNAs, the complementarity of 15-18 protospacer nucleotides may be sufficient to ensure efficient cleavage. Indeed, it has been shown that the disruption of complementarity in the 5'-terminal nucleotides of sgRNA not only does not reduce but even increases the efficiency of target hydrolysis [14,[115], [116], [117]]. More insight into this phenomenon was gained by analyzing the on-target and off-target activity of Cas9-sgRNAs using 5'-truncated guide RNAs (tru-gRNAs) [118]. Cas9 complexes loaded with tru-gRNAs possessing a 17- or 18-nucleotide guide sequence function as efficiently as those with a full-length 20-nucleotide sequence. Further shortening the targeting sequence of tru-gRNA to 16 nt or fewer resulted in the complete inhibition of RNP complex activity. Active truncated tru-gRNAs demonstrate high sensitivity to single- and double-nucleotide mismatches with the target sequence, thereby conferring high specificity to the system [119]. The targeting some genes with 18 nt sgRNAs in Drosophila also showed high efficiency and limited off-target effects [105]. While studying the in vivo cleavage of four different loci involving Cas9 and sgRNAs with reduced complementarity (18 nt and 17 nt instead of 20 nt) in yeast, a dramatic increase in hydrolysis specificity was observed [117]. However, the effect of sgRNA homology length on the specificity of target hydrolysis has not always been successfully reproduced under in vitro conditions. The results of target loci editing using tru-gRNA in various human cell types also display inconsistencies, likely due to variations in Cas9 expression levels under different intracellular conditions [120]. It has been postulated that the utilization of 5'-truncated sgRNAs has no appreciable effect on the stability of the dCas9 complex, indicating that effective cleavage in the case of tru-gRNA is not correlated with enhanced nuclease affinity [23]. These data align with observations from a study employing single-molecule Förster Resonance Energy Transfer (smFRET) to elucidate the conformational checkpoints of the Cas9-sgRNA-DNA complex [54]. In the presence of mismatched targets, the RNP complex containing a 17 nt guide sequence predominantly occupies its inactive conformation. This conformation serves as a “conformational checkpoint” for RNA-DNA hybrid complementarity. The energy barrier that regulates the transition of the HNH domain to its catalytically active state is sensitive to mismatches, thus introducing additional challenges for cleavage. These insights led M. Coelho and colleagues to develop a strategy to mitigate Cas9 off-target activity by blocking non-specific targets with truncated sgRNAs [121]. They proposed the use of dCas9 in tandem with a modified sgRNA, where 15 nucleotides of the guide sequence are fully complementary to an off-target locus. This configuration forms an inactive RNP complex that robustly binds to non-specific targets, thereby obstructing accessibility to fully active Cas9 and preventing cleavage.

Typically, the architecture of a 20-nt sgRNA guide sequence, transcribed via RNA polymerase, adheres to the 5'-GX19 pattern, with the guanine residue being crucial for transcription initiation [104,115]. Sequences conforming to the sgRNA context 5'-GGX_18_ have been identified as highly effective when employing T7 RNA polymerase, while the 5'-GAX_18_ and 5'-AGX_18_ contexts cause almost no mutations at the edited loci [107]. Moreover, augmenting the 5'-end of sgRNA with two additional guanine residues (5'-GGGX_19_) has been demonstrated to enhance hydrolysis specificity by improving discrimination against non-target sites [15]. This increased specificity is probably provided by the slower and more precise melting of the DNA duplex, which hinders R-loop stabilization in the presence of mismatches [122]. Thus, additional nucleotides at the 5'-end of the guide RNA may affect the frequency of mutations in target and non-target sites, potentially altering the stability, concentration, or secondary structure of sgRNA. It was also reported that truncating sgRNA from the 3'-end by a few nucleotides makes it more sensitive to mismatches in the target, thereby increasing editing specificity [19]. Another investigation has shown that extending the 3'-end of sgRNA improves the efficiency of target DNA editing in HEK293T cells [123]. The observed increase in efficiency may be attributed to the elevated expression levels of extended guide RNAs in the cell, which could result from their enhanced stability [21].

## 7 Conclusions and future perspectives

Efforts to create an "ideal" guide RNA with no off-target effects have significantly advanced the development of algorithms for predicting the off-target behavior of the CRISPR-Cas9 system. Optimal gene-editing tools necessitate a balance between high efficiency and specificity, which can often be at odds with one another (Table 1). A high efficiency of binding and cleavage at the target site, in many cases, correlates with a high probability of binding and cleavage at non-target sites [19]. The ideal balance between sensitivity to bona fide targets and potential off-target activity is dictated by the application. Early analyses of potential off-target sites have generated huge datasets of guide RNAs [17,19,21,104,107,124]. These findings resulted in the discovery of generally applicable rules governed by intrinsic features of the target site and sgRNA sequence. For example, the cutting frequency determination (CFD) score (also known as the “Doench rules”) considers both the position and identities of mismatched nucleotides [20]. This algorithm is based on cleavage data obtained for a large library containing thousands of guides targeting the CD33 gene for all PAMs, all possible nucleotide mismatches, and 1 bp indels at all positions. The MIT score (also known as “Hsu score”) estimates the off-target cleavage rate using a mismatch weight matrix derived from detailed studies of sgRNA variants [21]. A tool that accounts for sequences beyond simple mismatches uses the whole ensemble of active structures of the sgRNA-DNA complex, rather than a single structure [114]. More recent investigations used a free energy change estimated for nucleic acid duplexes to provide valuable parameters for Cas9 specificity and off-target assessment tools (Spec/SEAM-seq [89], CRISPRspec [110]). In particular, the parallel measurement of kinetic parameters for DNA binding and cleavage by SpCas9 provide mechanistic information about the effect of mismatches on cleavage, independent of their effect on affinity [89]. The free energy-based models for binding revealed that the penalty for mismatches within the seed region are not consistent among target sequences. The specificity correlates with the energy of exchange from a DNA-DNA duplex to an RNA-DNA heteroduplex. This indicates that binding, specifically the free-energy difference between on- and off-target binding, varies substantially between target sequences and is an important determinant of off-target cleavage. The process of R-loop formation by Cas9 is driven by free energy changes during the transition from DNA-DNA to RNA-DNA hybrid interactions [110]. This sequence-dependent free energy is largely determined by base-pairing and base-stacking energies. A guide mismatch tolerance (GMT) has been identified as characteristic of guide RNAs, which affects the R-loop formation [24,25,75,125]. Comparing GMT with the total base-stacking energy difference between DNA-DNA and RNA-DNA hybridization reveals distinct free energy landscapes for sgRNAs with high and low GMT [25]. The presence of a mismatch increases the energy barrier for R-loop formation, and the possibility of Cas9-sgRNA-DNA complex dissociation prior to target cleavage is determined by the relative height of these barriers. The further development of predictive technologies has expanded the range of parameters under consideration, including multiple mismatches, DNA bulges, RNA thermodynamics, DNA enthalpy and geometry, genomic locations, and gene expression level [22,101,126].

**Table 1:** Factors and mechanisms that can positively or negatively impact mismatch tolerance and specificity of Cas9.

Correlation	Factor	Reference
Positive	High concentration/expression level of Cas9	[19,116]
	Transition mismatches	[17]
	Mismatches within the DNA target (bubbles)	[50]
	1-2 nt PAM-distal mismatches	[3,4,16]
	Nucleotide ‘‘bulges’’ in the RNA-DNA heteroduplex	[20,69,75,100]
	Energetic stability of RNA-DNA vs. DNA-DNA duplex	[89]
Negative	RDR mismatches	[18,95]
	Controlling abundance of Cas9-sgRNA complex	[21,88]
	Thermodynamic stability of the guide RNA-DNA duplex.	[101,110]
	5'-truncated sgRNA (17–18 nt guide sequence)	[105,118,120]
	40–60 % G/C-content of guide sequence	[109]
	sgRNA extended by additional guanine residues (5'-GGGX_19_)	[15,122]
	Engineered Cas9 variants, dual Cas9	[55,89,94,116,127]

In vivo, Cas9 encounters the entire genome, and potential gRNA targets are relatively rare. The success of genome editing in living cells depends on several factors, including the method of RNP complex delivery, efficiency of the Cas9 expression and sgRNA maturation, the lifetime of the active complex in specific cellular environments, the accessibility of target loci within chromosomal contexts, etc. By directing the dCas9 to genomic DNA of *Streptococcus pneumoniae,* it was found that the tolerance to mismatches in RNA-DNA hybrids is greater under in vitro than in vivo conditions [128]. However, comparative analysis of Cas9-sgRNA specificity in yeast indicates similar mismatch tolerance profiles in terms of mismatch position and the lengths of guide sequence in vitro and in vivo [117]. The prolonged incubation of the RNP complex with target DNA in vitro results in a notable off-target cleavage of sequences containing 1–2 mismatches in the PAM-distal region. High-throughput sequencing suggests that some of the off-target sites cleaved by Cas9 complexes in vitro are also hydrolyzed in living cells, and off-targets with the highest frequency in vitro are most frequently detected in vivo [19]. Comparisons of RNP complex activity in mammalian and yeast cells show more overlaps than differences, suggesting that determinants of Cas9 specificity are generally shared across cellular environments [117]. Reported discrepancies in off-target activity levels and editing efficiency across cell types likely arise from variations in intracellular conditions [14,69], RNP complex delivery methods, and target DNA accessibility. Chromatin accessibility is considered a major determinant of the DNA-binding capacity of the RNP complex [88]. In HEK293T cells, modified dCas9 exhibits more active interactions with promoters and 5′-untranslated region (UTR) exons than with intergenic regions [83]. The preferential enrichment of off-targets in accessible chromatin has implications for modulation of transcriptome using dCas9. As mentioned by X. Wu and colleagues [10], regulatory elements of active genes, such as promoters and enhancers, are significantly enriched for off-target binding since those elements are accessible when active. Additionally, off-target activity appears to depend on DNA packing density. Distortion of the DNA duplex structure under severe stretching leads to numerous non-specific binding events [129]. DNA with high levels of negative supercoiling can be cleaved by the Cas9-sgRNA complex despite a few PAM-distal mismatches, whereas torsionally relaxed DNA undergoes more specific cleavage [130]. Therefore, even with optimal guide sequence design rules, certain cellular conditions can make some genes difficult to edit [69,104]. Off-target cleavage is multifactorial, and a comprehensive understanding of the specificity of a given sgRNA–target pair will require biochemical analyses of off-target candidates followed by detailed cellular studies.

The SpCas9 protein demonstrates a degree of tolerance to guide RNA sequences containing up to five mismatches. However, orthologous nucleases from other species, such as *Staphylococcus aureus*, *Neisseria meningitides*, *Campylobacter jejuni*, and *Streptococcus equinus*, exhibit reduced off-target effects, thus enhancing their specificity [79,80,131,132]. The variation in PAM recognition requirements among these orthologs expands the repertoire of potential genomic editing sites [76]. A significant advancement in increasing the specificity and reducing mismatch tolerance of Cas9 was the generation of engineered variants with specific amino acid substitutions. In particular, mutations introduced into the REC domain for the SpCas9-HF1 and eSpCas9 variants [133,134], when bound to non-target sequences, prevent the conformational transitions required for the HNH domain activation [58]. Some mutations lead to loosening interactions between the RNP complex and the target during RNA-DNA hybrid formation, thereby increasing the requirement for precise complementarity between sgRNA and the target [89]. Directed bacterial evolution techniques have further expanded the arsenal of highly accurate Cas9 homologs [135,136]. The off-target activity in the CRISPR-Cas9 system is generally viewed as a detrimental effect that requires mitigation. Nevertheless, recent studies have suggested that a certain level of mismatch tolerance can be advantageous in particular genetic engineering applications. For instance, Cas9 complexes with 5'-truncated sgRNA have been effectively employed as negative selection tools to target single-nucleotide mutations within bacterial genomes [137]. The high specificity of the RNP complex with tru-gRNA prevents the editing of sequences that differ from the protospacer by even a single nucleotide. Consequently, cells containing fully complementary target DNA undergo apoptosis due to induced double-strand breaks, while mutant cells, which do not align with the guide RNA, remain unaffected. Another strategy leveraging single-nucleotide mismatches aims to enhance allele-specific genome editing, particularly in situations where a pathological condition arises from a gene mutation on one allele. By designing a truncated guide RNA that mismatches the pathogenic allele by one nucleotide and the normal allele by two nucleotides, cleavage is directed exclusively at the mutant allele, enabling selective genomic DNA modification [25].

Today, many biochemical screening techniques for off-target site identification are available. The methods based on next-generation sequencing, like GUIDE-seq [50], HTGTS [138], Digenome-seq [52], CIRCLE-seq [69], SITE-seq [51], and CHANGE-seq [139], are considered the gold standard for validating off-targets in vitro and in vivo. Datasets of experimentally validated true off-target sites derived from genome-wide assays form the basis for computational approaches that use alignment, hand-crafted rules, and machine learning for prediction of off-target sites (MIT [21], Elevation [22], CCTop [140], CRISPR-HW [141], CRISTA [126], DeepCRISTL [142], etc.). All the techniques have their individual strengths and limitations (reviewed in Ref. [143]). Despite the high sensitivity of these methods, the detection of ultra-low off-target levels provided by engineered Cas9 variants is still not sufficiently precise. In particular, the detection limit for amplicon-based high-throughput sequencing is 0.1 % due to its inherent error rate, resulting in false positives. It might be insufficient to identify all potentially dangerous off-target editing events, especially under prolonged Cas9 expression. Computational methods always employ existing off-target cleavage data (with tens of thousands of guide sequences tested) as a training set to predict and rank potential off-target sites [21,144]. However, the accuracy of these algorithms is not ideal, since datasets of experimentally validated true off-target sites are limited. X. R. Bao et al. sequentially assessed the performance of the existing experimental and computational methods. The challenge is that the datasets generated by disparate gRNA sequences in disparate experimental systems are not all directly comparable. In addition, many methods are performed on purified DNA, which lacks the chromatin structure that can potentially prevent cutting by programmable nucleases within cells. Nevertheless, GUIDE-seq has shown most promising results for the detection of off-targets sites, with the highest on-target enrichment and a moderate number of false positives. Some computational methods do not consider DNA/RNA bulges or have no ability to rank off-target sites, yet show remarkable agreement for predicting off-target sites at which editing rates are high. The divergence increases at sites with lower off-target editing rates. Based on this comparison, Elevation and CRISTA were included in the category of the most accurate prediction models. In practical applications, the authors recommend using at least one bioinformatics-based tool and one experimental tool to decrease the possibility of missing true off-target editing. The false-positive and false-negative rates demonstrated by existing methods may be acceptable for many research-oriented applications but pose potential risks for therapeutic applications where large numbers of patient cells are involved. Plentiful future research will be required to determine what the detection limit should be and further technological development will be needed to achieve this. The constant improvement in the activity and specificity of the latest engineered Cas9 variants has led to off-target levels that would not be measurable with existing detection tools. Enhancing the sensitivity and accuracy of off-target detection techniques is crucial for the safe and effective integration of CRISPR/Cas9 genomic editing technologies into both biotechnological research and therapeutic practices.

## CRediT authorship contribution statement

**Lyubov Yu. Kanazhevskaya:** Writing – review & editing, Visualization, Writing – original draft, Investigation. **Polina V. Zhdanova:** Writing – original draft, Software, Writing – review & editing, Visualization, Project administration. **Alexander A. Chernonosov:** Writing – review & editing, Visualization, Supervision, Conceptualization, Writing – original draft, Validation, Investigation. **Vladimir V. Koval:** Writing – original draft, Project administration, Writing – review & editing, Supervision, Conceptualization.

## Funding

This research was partially supported by the Russian Science Foundation (grant no. 20-14-00214), by the Russian Ministry of Science and Higher Education (agreement No. 075-15-2022-263), and by a Russian state-funded project for the ICBFM SB RAS (grant No. 121031300056-8).

## Declaration of competing interest

The authors declare that they have no known competing financial interests or personal relationships that could have appeared to influence the work reported in this paper.
